# δ-Catenin Activates Rho GTPase, Promotes Lymphangiogenesis and Growth of Tumor Metastases

**DOI:** 10.1371/journal.pone.0116338

**Published:** 2015-01-30

**Authors:** Sampa Ghose, Yongfen Min, P. Charles Lin

**Affiliations:** 1 Center for Molecular Medicine, Jawaharlal Nehru University, New Delhi, India; 2 Mouse Cancer Genetics Program, Center for Cancer Research, National Cancer Institute, Frederick, Maryland, 21702, United States of America; Feinberg Cardiovascular Research Institute, Northwestern University, UNITED STATES

## Abstract

δ-catenin, an adherens junctions protein, is not only involved in early development, cell-cell adhesion and cell motility in neuronal cells, but it also plays an important role in vascular endothelial cell motility and pathological angiogenesis. In this study, we report a new function of δ-catenin in lymphangiogenesis. Consistent with expression of δ-catenin in vascular endothelial cells, we detected expression of the gene in lymphatic endothelial cells (LECs). Ectopic expression of δ-catenin in LECs increased cell motility and lymphatic vascular network formation *in vitro* and lymphangiogenesis *in vivo* in a Matrigel plug assay. Conversely, knockdown of δ-catenin in LECs impaired lymphangiogenesis *in vitro* and *in vivo*. Biochemical analysis shows that δ-catenin regulates activation of Rho family small GTPases, key mediators in cell motility. δ-catenin activates Rac1 and Cdc42 but inhibits RhoA in LECs. Notably, blocking of Rac1 activation impaired δ-catenin mediated lymphangiogenesis in a Matrigel assay. Consistently, loss of δ-catenin in mice inhibited the growth of tumor metastases. Taken together, these findings identify a new function of δ-catenin in lymphangiogenesis and tumor growth/metastasis, likely through modulation of small Rho GTPase activation. Targeting δ-catenin may offer a new way to control tumor metastasis.

## Introduction

Lymphangiogenesis is the process that forms lymphatic vessels, during which lymphatic endothelial cells proliferate, migrate and assemble into lymphatic vascular networks. Lymphangiogenesis plays important roles in tissue homeostasis, metabolism and immunity. Lymphatic vessel formation also contributes to pathological conditions such as tumor invasion to lymph nodes and metastasis [1]. The role of the lymphatic network in human diseases has received renewed interest largely due to the identification of specific signaling pathways that regulate the formation of lymphatic systems, such as vascular endothelial growth factor C (VEGF-C) and its cognate receptor VEGF receptor 3 (VEGFR-3). VEGF-C stimulates tumor lymphangiogenesis and metastasis by interacting with VEGFR3 [1].

δ-catenin is a member of the catenin family belonging to the p120 catenin (p120ctn) subfamily [2]. The major functions of the p120 protein family include stabilization of cadherins by binding to a highly conserved sequence in the juxtamembrane region, as well as regulating RhoGTPase activity and cell motility [2]. δ-catenin is expressed in the brain where it is important for normal cognitive development. Similar to other catenins, δ-catenin regulates cell-cell adhesion and cell motility [3]. Genetic deletion of δ-catenin in mice results in severe deficits in several types of memory as well as synaptic plasticity [4], consistent with the neuronal phenotype. As molecular mechanisms underlying the development of the nervous system have been co-opted by the vasculature and certain neuronal guidance molecules also regulate angiogenesis and vascular patterning [5,6], we reported that δ-catenin is also present in vascular endothelium. It regulates vascular endothelial cell motility and pathological angiogenesis [7].

To further explore the function of δ-catenin in adult vasculature, we investigated the role of this protein in lymphangiogenesis. We detected expression of δ-catenin in lymphatic endothelial cells. δ-catenin regulates lymphangiogenesis *in vitro* and *in vivo*, likely through regulating small RhoGTPase activities. Consistently, inactivation of δ-catenin resulted in a significant reduction of lymphangiogenesis and growth of tumor metastases in mouse models. Thus, this study expands out knowledge and identifies a new mediator in δ-catenin in lymphangiogenesis in tumor progression.

## Material and Methods

### Mice and cell lines

The mice were maintained in pathogen-free facilities at Vanderbilt University and the National Cancer Institute (Frederick, MD). The study was approved by the Vanderbilt University Animal Care and Use Committee and the NCI Animal Care and Use Committee, and in accordance with the ARRIVE guidelines. δ-catenin null mice on a C57/Bl6 background were used in our previous study [7]. δ-catenin heterozygous null mice were used for breeding to generate wild type and homozygous null mice. Age and sex matched littermate mice were used in each study. The animal study only involve tumor cell injection, no step is needed to ameliorate suffering. At the end of the experiment, the mice were euthanized by CO_2_ asphyxiation.

Human lung lymphatic endothelial cells (HMVEC-LLy) were purchased from Lonza (Walkersville Inc.), and cultured according to manufacture’s protocol. All the experiments were performed on cells between 3–7 passages. Lewis lung adenocarcinoma cells (3LL) were maintained in DMEM supplemented with 10% serum.

### Isolation of murine lymphatic endothelial cells

Eight weeks old of wild type and δ-catenin null mice were sacrificed. Single cell suspension was made from lungs as described [8], followed by incubation with Lyve-1-PE antibody from MBL. Positive cells were sorted in a FACStar Plus flow cytometer (Becton Dickinson, Franklin Lakes, NJ). Cell purity was confirmed by FACS with a Lyve-1 antibody. Cells with over than 95% purity were used in studies.

### RT-PCR and Western blot

Total RNA was isolated by RNeasy Mini kit (Qiagen, CA). iScriptcDNA Synthesis kit (Bio-Rad) was used to make cDNA, followed by RT-PCR using specific primer sets. Murine δ-catenin, 5’-GCCCAGTTTGAGAAGCTG 3’ and 5’-TCCTCGGTGTAGGTTTCC-3’; Βeta-actin 5’- GACAACGGCTCCGGCATGTGC -3’ and 5- TGGCTGGGGTGTTGAAGGTC- 3’.

Cell lysates were analyzed by Western blot and incubated with a specific antibody against δ-catenin (Santa Cruz).

### Ectopic expression and knockdown of δ-catenin in LECs

HMVEC-LLy cells were transiently transfected with either a δ-catenin expression pCMV vector or empty vector using a Nucleofector device (Lonza). Knockdown of δ-catenin was performed by using a mixture of shRNA (Sigma-Aldrich) transfected into cells by a Nucleofector device (Lonza).

*In vitro* lymphatic angiogenic assays and Rac1, Cdc42, and RhoA activation assay.

LEC migration, lymphatic vascular network formation and Rho GTPase activity measurement were done according to a published protocol except using VEGF-C [7]. The experiments were done in triplicate and repeated three times.

### Lymphangiogenesis and tumor metastasis model

For lymphangiogenesis, 600 μl of growth factor reduced Matrigel (BD Bioscience) mixed with VEGF-C at 100 ng was injected subcutaneously into the dorsal region of sex and age matched mice for about 2 weeks. Matrigel plugs were harvested, sectioned and analyzed by Lyve-1 immunofluorescent staining. The number of Lyve-1 positive vascular structures was counted from 10 randomly selected fields under microscopy. For metastasis, 3LL cells (1x10^5^) in 100μl PBS were injected into the tail vein of wild-type littermates and δ-catenin null mice. At 14 days after inoculation of the tumor cells, the lungs were excised, and the number of pulmonary tumor colonies was counted and lungs were weighted.

### Statistical analysis

The results are represented as means ± SE for each group. The statistical significance of difference between two groups was calculated by student’s *t* test. Difference were considered statistically significant if p< 0.05.

## Results

### δ-catenin is expressed in LECs, and it regulates cell migration and lymphangiogenesis likely through its effects on RhoGTPase *in vitro*

**Figure 1:**
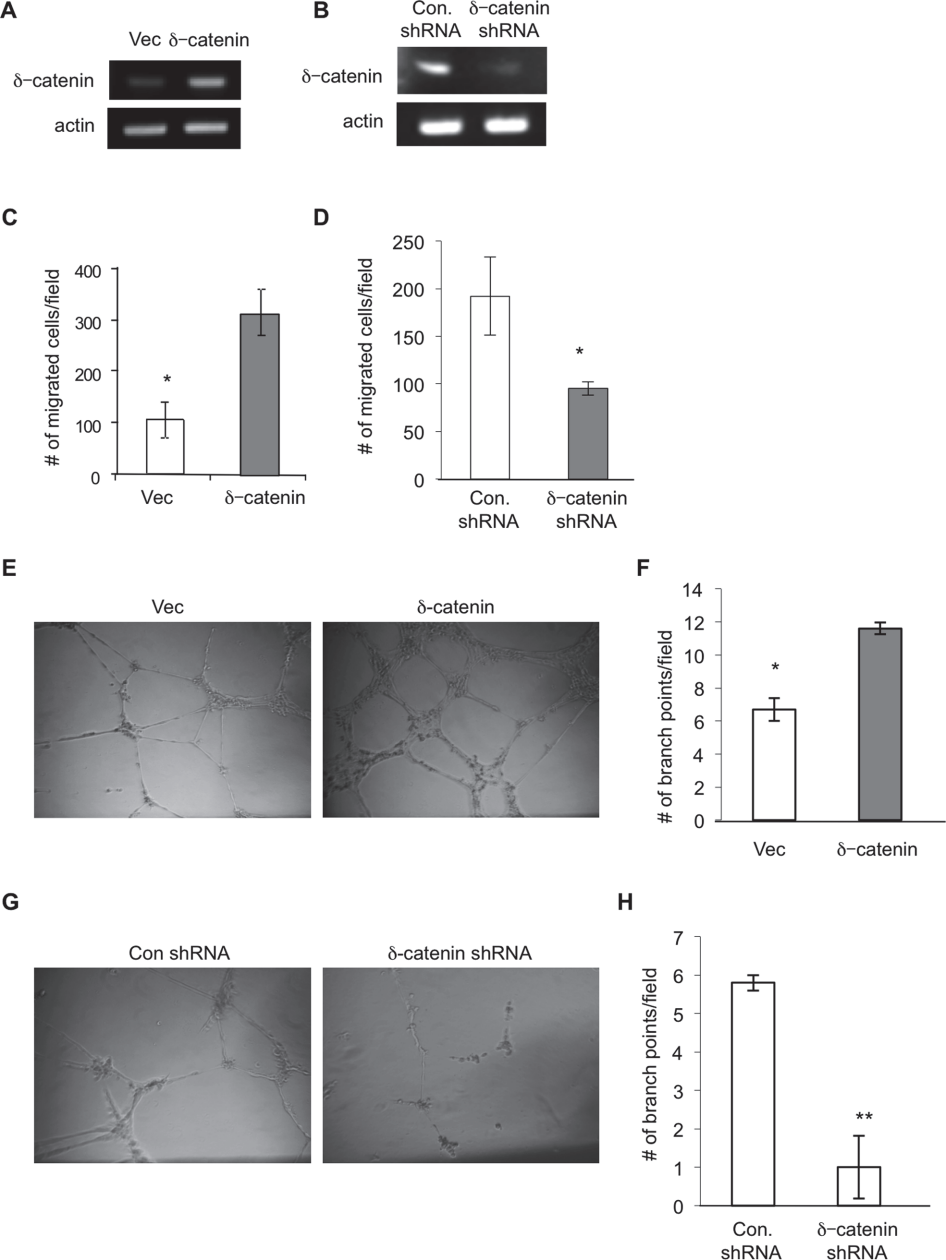
δ-catenin is present in lymphatic endothelial cells and regulates lymphangiogenesis *in vitro*. HMVEC-LLy cells were transfected with either an empty vector (vec) or an expression vector for δ-catenin for 48 hours. Total RNA was isolated and δ-catenin mRNA levels were quantitated by semi quantitative RT-PCR (Panel A). HMVEC-LLy cells were either transfected with a mixture of δ-catenin specific shRNA constructs or control shRNA construct for 48 hours, respectively. Total RNA was isolated and δ-catenin mRNA levels were quantitated by semi quantitative RT-PCR (Panel B). Cell migration was measured in a Transwell chamber by seeding HMVEC-LLy cells transfected with empty vector or δ-catenin expression vectors. Migrated cells were counted from 10 randomly selected high power fields under microscopy 4 hours after incubation and graphed with mean and standard error (Panel C) *p<0.05. Cell migration was also measured by seeding δ-catenin knockdown HMVEC-LLy cells or control shRNA transfected cells for 4 hours, followed by the same procedure as described above (Panel D) *p<0.05. HMVEC-LLy cells transfected with either a vector control or δ-catenin expression vector were incubated on Matrigel for 24 hours. The images of vascular network formation were taken under microscopy (Panel E). The number of vascular branch points was counted in 10 randomly selected fields under microscopy (Panel F). Vascular network formation in Matrigel was evaluated using δ-catenin knockdown HMVEC-LLy cells and control cells (Panel G and H). Representative images were shown. *p<0.05; **p<0.01. Each experiment was done in triplicate and the experiment was repeated twice.

Based on our previous report that δ-catenin is expressed in vascular endothelial cells and regulates pathological angiogenesis, we postulated a similar role of the gene in lymphangiogenesis. To test this hypothesis, we initially determined if the gene is expressed in LECs by using primary human lung lymphatic microvascular endothelial cells (HMVEC-LLy) pooled from multiple donors. Semi quantitative RT-PCR analysis clearly demonstrated expression of δ-catenin in human lymphatic endothelial cells (Fig. 1A). To further explore its role in lymphangiogenesis, we either ectopically expressed δ-catenin (Fig. 1A) or knocked down the gene in HMVEC-LLy (Fig. 1B), followed by examining the function of δ-catenin in lymphangiogenesis. We found that ectopic expression of δ-catenin significantly increased cell motility and conversely knockdown of δ-catenin expression significantly inhibited cell motility in response to VEGF-C stimulation in a Transwell cell migration assay (Fig. 1C and 1D).

Like vascular endothelial cells, lymphatic endothelial cells have the tendency to form vascular networks in 3-D culture. Consistent with the increase in cell motility, ectopic expression of δ-catenin significantly increased lymphatic vascular network formation (Fig. 1E and 1F) and conversely knockdown of δ-catenin expression significantly inhibited the process in a Matrigel assay when compared to vector controls (Fig. 1G and 1H). Notably, we did not observe any significant difference on cell proliferation between the groups by counting the number of live cells in a period of several days in culture (data not shown). These results identify a new regulator in δ-catenin in lymphangiogenesis. δ-catenin is expressed in lymphatic endothelial cells, and it regulates lymphangiogenesis likely through its effect on cell motility, an integral step in lymphangiogenesis.

**Figure 2:**
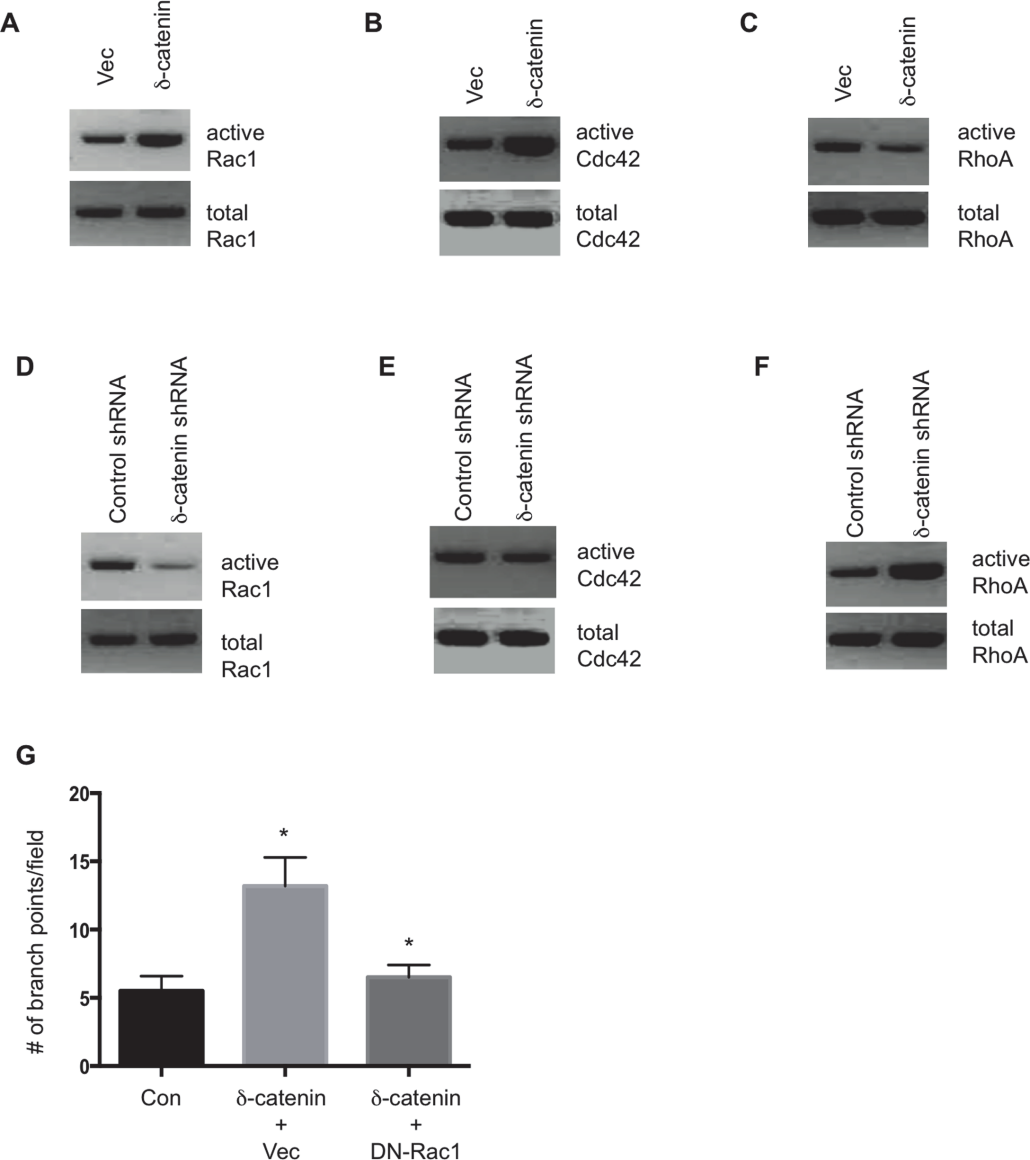
δ-catenin regulates Rho GTPase activity in LECs. HMVEC-LLy cells were transfected with an empty vector or δ-catenin expression vectors for 48 hours. Cell lysate was collected and analyzed for activation of Rac1 (Panel A), Cdc42 (Panel B), and RhoA (Panel C). Activation of Rac1 and Cdc42 was determined by using a PAK1- pull-down assay while active RhoA was pulled down with Rhotekin-agarose beads. For knockdown experiments, HMVEC-LLy cells were transfected with shRNA for δ-catenin or control shRNA constructs for 48 hours. Cell lysate was collected and analyzed for activation of Rac1 (Panel D), Cdc42 (Panel E), and RhoA (Panel F) by Western blotting. HMVEC-LLy cells transfected with a vector control or δ-catenin expression vector plus a vector control or Rac1N17 were incubated on Matrigel for 24 hours. The number of vascular branch points was counted in 10 randomly selected fields under microscopy (Panel G). All experiments were repeated three times. Mean and SE were plotted. * p<0.05.

Small Rho family of GTPase regulates cell motility. Previously, it was reported that δ-catenin regulates neuronal cell motility through Rho GTPase [9]. We show a similar mechanism of δ-catenin in regulating vascular endothelial cell motility [7]. We therefore determined the effects of δ-catenin on RhoGTPase activity in LECs. We ectopically expressed δ-catenin in HMVEC-LLy cells, followed by a measurement of RhoGTPase activation. The results show that δ-catenin activates Rac1 and Cdc42 (Fig. 2A and 2B), but inhibits RhoA activation (Fig. 2C). Conversely, knockdown of δ-catenin in LECs inhibited Rac1 and CDC42 activity, but increased RhoA activity (Fig. 2D–2F).

To validate if the RhoGTPase is responsible for δ-catenin mediated lymphangiogenesis, we co transfected HMVEC-LLy cells with vectors for δ-catenin and dominant-negative Rac1 (Rac1N17). As expected that blocking Rac1 activation significantly inhibited δ-catenin-mediated lymphangiogenesis in a Matrigel tubule formation assay (Fig. 2G). These data suggest that δ-catenin regulates lymphatic endothelial cell motility and lymphangiogenesis likely through a regulation of small Rho GTPase activities.

### δ-catenin regulates lymphangiogenesis and growth of tumor metastases *in vivo*

**Figure 3:**
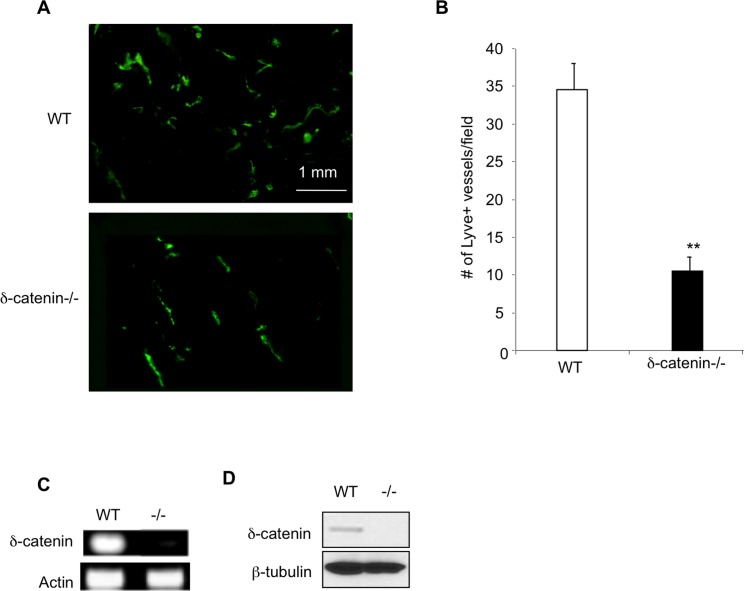
Deletion of δ-catenin in mouse impairs lymphangiogenesis *in vivo*. Recombinant VEGF-C at 100 ng was mixed with 100 μl of ice cold Matrigel, and injected sc into the dorsal skin region in age and sex matched wild type and δ-catenin null mice (2 injections per mouse). The solidified Matrigel plus was harvested in 2 weeks after the implantation, sectioned and stained with an anti Lyve-1 antibody (Panel A). The number of Lyve-1 positive vascular structures was counted in 10 randomly selected fields under microscopy and graphed (Panel B). n = 8 gel plugs per group. **p<0.01. Murine lung lymphatic microvascular endothelial cells were isolated and purified from pooled 7 weeks old wild type and δ-catenin null mice (n = 4 mice per group) by flow cytometry using antibody against Lyve-1. Total RNA was isolated and subjected to RT-PCR with δ-catenin specific primers (Panel C). Cell lysates were analyzed by Western blot and incubated with an antibody specific for δ-catenin (Panel D).

To further explore the function of δ-catenin in lymphangiogenesis *in vivo*, we used δ-catenin null mice. Besides deficiency in memory and synaptic plasticity, the null mice are healthy, fertile, and they grow normally [4]. We established a Matrigel plug assay, in which we mixed recombinant VEGF-C with growth factor depleted Matrigel, followed by implantation of the gel subcutaneously in WT and δ-catenin null mice. Matrigel plugs were harvested 2 weeks later, sectioned and incubated with an antibody against Lyve-1, a marker for lymphatic endothelium (Fig. 3A). Quantification of Lyve-1+ lymphatic vessels in the gel plugs confirmed a significant reduction of lymphangiogenesis associated with δ-catenin null mice when compared to WT littermate controls (Fig. 3B). To ensure defective lymphangiogenesis in δ-catenin null mice is due to a direct effect of the gene in lymphatic endothelial cells, we purified lymphatic endothelial cells from lungs of wild type and δ-catenin knockout mice using Lyve-1 antibody conjugated with magnetic beads. Flow cytometry analysis confirmed over than 95% purity of Lyve-1 positive cells. We then examined expression of δ-catenin from these samples using semi quantitative RT-PCR. Consistent with the data in human lymphatic endothelial cells, we detected expression of δ-catenin transcripts in wild type LECs, but not in the null cells (Fig. 3C). In addition, we confirmed δ-catenin protein expression in murine LECs using Western blot (Fig. 3D). These data implicate broad expression of δ-catenin in lymphatic endothelial cells. It regulates cell motility and lymphangiogenesis.

**Figure 4:**
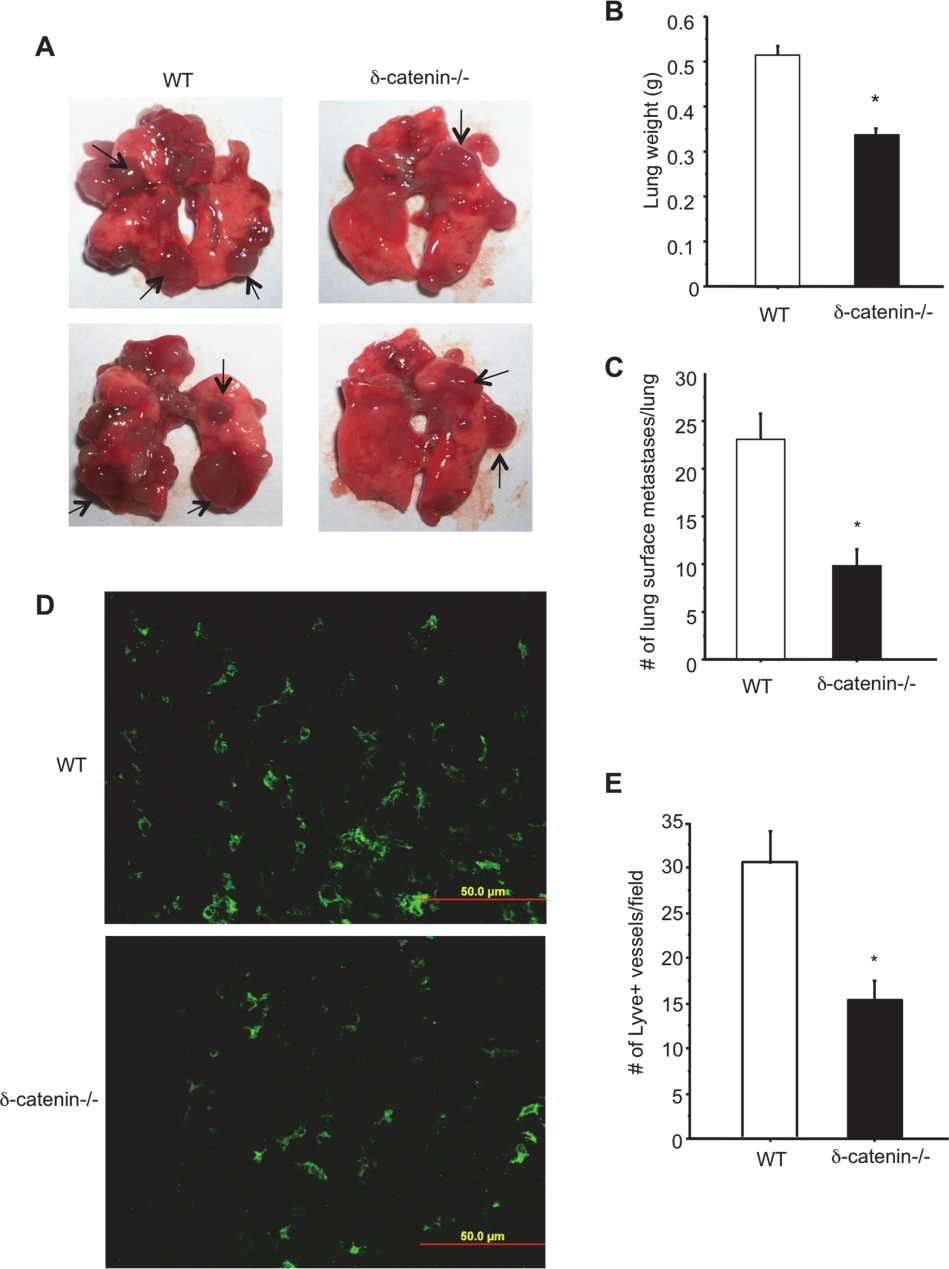
Loss of δ-catenin impairs lymphangiogenesis and tumor growth in an experimental metastasis mouse model. 3LL cells (1x10^5^) were injected into tail vein of wild type littermates and δ-catenin null mice. Two weeks after the injection, lungs were excised from mice and images were taken (Panel A). Arrows point to tumor metastases in lungs. Lung weight (Panel B) and lung surface metastases (Panel C) were quantified and graphed. *p<0.05. Tumor metastases were sectioned and stained with an antibody against Lyve-1 (Panel D). Lyve-1 positive vascular structures were counted in 10 randomly selected high power fields under microscopy and graphed (Panel E). *p<0.05. Representative images were shown. This experiment was repeated twice and each time 5 mice per group were used.

As lymphatic vessels contribute to tumor metastasis, we next examined the function of δ-catenin in tumor growth using an experimental vascular metastasis model. We injected 3LL Lewis lung carcinoma cells into the tail vein of δ-catenin null and wild type littermate control mice for two weeks, followed by evaluation of lung metastasis. Gross examination of lungs showed a reduction of tumor metastases in the null mice compared to wild type controls (Fig. 4A). Quantitation of lung metastasis confirmed the finding. There is a significant reduction of metastasis measured by either lung weight (Fig. 4B) or counting lung surface metastases (Fig. 4C) in the δ-catenin null mice. The tumor tissues were harvested, processed and sectioned. An examination of tissue sections of lung metastases by immuno fluorescent staining using an antibody against Lyve-1 confirmed a significant reduction of tumor lymphangiogenesis in the null mice compared to controls (Fig. 4D and 4E). Taken together, these *in vitro* and *in vivo* findings clearly established an important role of δ-catenin in lymphangiogenesis. Loss of δ-catenin in mice impairs lymphangiogenesis and consequently contributes to reduced growth of tumor metastases.

## Discussion

The spread of cancer cells, either by local invasion or distant metastasis, is a hallmark of malignancy. The majority of cancer-associated death is due to metastasis. The lymphatic system is important for the metastatic spread of cancer. In this study, we identified a new mediator in δ-catenin in lymphangiogenesis and growth of tumor metastases. δ-catenin is expressed in lymphatic endothelial cells. It regulates cell motility, lymphatic vascular network formation *in vitro* and lymphangiogenesis *in vivo*, likely through regulating Rho GTPase activity. Consequently, deletion of δ-catenin in mice impaired growth of tumor metastases in a murine vascular metastasis model. This finding plus our previous data of δ-catenin in pathological angiogenesis [7] suggest δ-catenin has broad functions in vascular biology. δ-catenin is a promising molecular target for cancer treatment.

δ-catenin was originally identified as a neuronal catenin [9]. It is localized at the post-synaptic adherens junction, collaborates with Rho GTPases to set a balance between neurite elongation and branching, and induces dendritic protrusions in neurons [3]. The vascular system and the sensory nerve system have a similar morphology and share common cues in regulation of the morphogenesis of both systems [10], resulting in a branching pattern that mirrors each other. Consistent with this notion, we reported expression of δ-catenin in the vascular endothelium [7]. The lymphatic and blood vascular systems serve distinct yet complementary functions to maintain tissue homeostasis. Lymphatic endothelial cells are derived from venous endothelial cells in development. It is not surprising that we detected expression of δ-catenin in lymphatic endothelial cells in the current study.

δ-catenin belongs to the p-120ctn sub family. It is well known that p120ctn regulates actin cytoskeleton organization and cell motility through Rho family GTPase [11]. Similarly, δ-catenin regulates cell motility in neural cells [9], vascular endothelial cells [7], as well as lymphatic endothelial cells as reported in this study. Expression of p120ctn disrupts stress fibers and focal adhesions and results in an increased activity of Cdc42 and Rac1, and a decrease in RhoA activity. The p120ctn-induced phenotype is blocked by dominant negative Cdc42 and Rac1 and by constitutively active Rho-kinase, but is enhanced by dominant negative RhoA [12]. Consistently, we found that ectopic expression of δ-catenin in lymphatic endothelial cells activates Rac1 and Cdc42, and inhibits RhoA activity, and conversely, knockdown of δ-catenin in cells inhibits Rac1 and Cdc42 activity with increases RhoA activity. We previously reported that Vav1, a guanine nucleotide exchange factor for Rho GTPase, directly interacts with δ-catenin and is responsible for δ-catenin mediated Rac1 and Cdc42 activity in vascular endothelial cells [7]. Since Vav1 is expressed in hematopoietic cells and endothelial cells, it is conceivable to suggest that Vav1 may possess a similar function in lymphatic endothelial cells.

In conclusion, this study identifies a new function of δ-catenin in lymphangiogenesis. δ-catenin is expressed in lymphatic endothelial cells. It regulates cell motility and vascular network formation likely through regulation of Rho GTPase signaling. Consistently, inactivation of δ-catenin in mouse models inhibits lymphangiogenesis and growth of tumor metastases. The study expands our understanding of δ-catenin in vascular biology. Considering the critical function of δ-catenin in pathological angiogenesis [7] and lymphangiogenesis, targeting δ-catenin may offer a new avenue for cancer therapy.

### Ethical Standards

The experiments comply with the current laws of USA in which they were performed.
